# Sports-related sudden cardiac death due to myocardial diseases on a population from 1–35 years: a multicentre forensic study in Spain

**DOI:** 10.1080/20961790.2019.1633729

**Published:** 2019-08-19

**Authors:** Benito Morentin, M. Paz Suárez-Mier, Ana Monzó, Pilar Molina, Joaquín S. Lucena

**Affiliations:** a Section of Forensic Pathology, Basque Institute of Legal Medicine, Bilbao, Spain;; b Histopathology Service, Department of Madrid, National Institute of Toxicology and Forensic Sciences, Spain;; c Forensic Pathology Service, Institute of Legal Medicine and Forensic Sciences, Valencia, Spain;; d Forensic Pathology Service, Institute of Legal Medicine and Forensic Sciences, Seville, Spain

**Keywords:** Forensic sciences, forensic pathology, sudden cardiac death, sports, myocardial diseases, autopsy, young

## Abstract

This is a multicentre forensic study that identifies all sports-related sudden deaths (SRSDs) in young people, due to myocardial diseases (MDs) that occurred in a large area of Spain. The aim of the study is to assess the epidemiology, causes of death, and sport activities associated with these fatalities. This is a retrospective study based on forensic autopsies performed in the provinces of Biscay, Seville, Valencia and in the jurisdiction covered by the National Institute of Toxicology and Forensic Sciences in Madrid (Spain). The retrospective study encompasses from 2010 to 2017. All sudden cardiac deaths (SCDs) in persons 1–35 years old were selected. The total number of SCDs were divided into death occurred during exercise (SRSD) and death during rest, sleep or normal activities (non-SRSD). Each of these two groups was subdivided according to the cause of death into MD (primary cardiomyopathies and myocarditis) and non-MD. Clinic-pathological, toxicological and genetic characteristics of SRSD due to MD were analysed. Over the 8-year study period, we identified 645 cases of SCD in the young: 75 SRSD (11.6%) and 570 non-SRSD (88.4%). MD was diagnosed in 33 (44.0%) of the SRSD and in 112 (19.6%) of the non-SRSD cases. All cases of SRSD due to MD were males (mean age (24.0 ± 7.6) years) practicing recreational sports (85%). SRSDs were more frequent in arrhythmogenic cardiomyopathy (ACM) (37%) and hypertrophic cardiomyopathy (HCM) (24%), followed by myocarditis (15%) and idiopathic left ventricular hypertrophy (ILVH) (9%). Only in five cases of SRSD the MD responsible of death (HCM) had been diagnosed in life. Cardiovascular symptoms related to the disease were present in other seven patients (six of them with ACM). Postmortem genetic studies were performed in 15/28 (54%) primary cardiomyopathies with positive results in 12 (80%) cases. The most frequent sports disciplines were football (49%) followed by gymnastics (15%) and running (12%). In Spain, SRSD in young people due to MDs occurs in males who perform a recreational activity. Compared with control group we observed a strong association between MDs and exertion. One in three SRSDs are due to cardiomyopathy, especially ACM, which reinforces the need for preparticipation screening to detect these pathologies in recreational sport athletes. Further studies are warranted to understand the causes and circumstances of sudden death to facilitate the development of preventive strategies.

## Introduction

The benefits of regular sports activity to prevent a cardiovascular event are well recognized in medical literature. However, in people with concealed cardiovascular disorders, the risk of sudden cardiac death (SCD) during exertion is increased [1]. The prevalence of sports-related sudden death (SRSD) is higher in adults than in young people, with the majority of them due to an underlying cardiovascular disease, which usually has not been diagnosed during life, mainly coronary atherosclerotic disease. Although the occurrence of a sudden death (SD) during the participation in sports activities is a very rare episode, these deaths have a dramatic impact in families as well as in general and medical communities, especially if the victim is an elite competitive athlete.

In the last decades several researches have investigated SRSD in adolescents and young people. It has been estimated that sports activity in this age group is associated with approximately 2.5- to 4.5-fold greater risk of SCD being the incidence approximately of 0.46 cases per 100 000 person-year or 2.3 per 100 000 athletes per year [2–4]. Causes of death differ notoriously with regard to cohorts (general population, competitive athletes or military personnel) and to the geographical region of the study. Myocardial diseases (MDs) are one of the main causes of death in persons ≤35 years with frequencies ranging from 25% to 62% [5–11]. However, population-based studies on SRSD during competitive and recreational sports are scarce.

Forensic investigations based on autopsies are useful to improve our knowledge on the aetiology, circumstances and precipitating factors of SRSD, and to develop effective strategies including pre-participation screening and availability of external automatic defibrillators (EADs) that could help to prevent these dramatic events [12].

Taking into consideration this background, we carried out a multicentre forensic study in the general population with the main aim to assess the epidemiology, clinic-pathological characteristics, causes of death, and sports activities associated with SRSD due to MD in 1**–**35-year-old people. As a secondary objective we wanted to know if the characteristics and causes of death due to MD are different or not in SRSD and non-SRSD.

## Methods

### Study design

**Figure 1.**
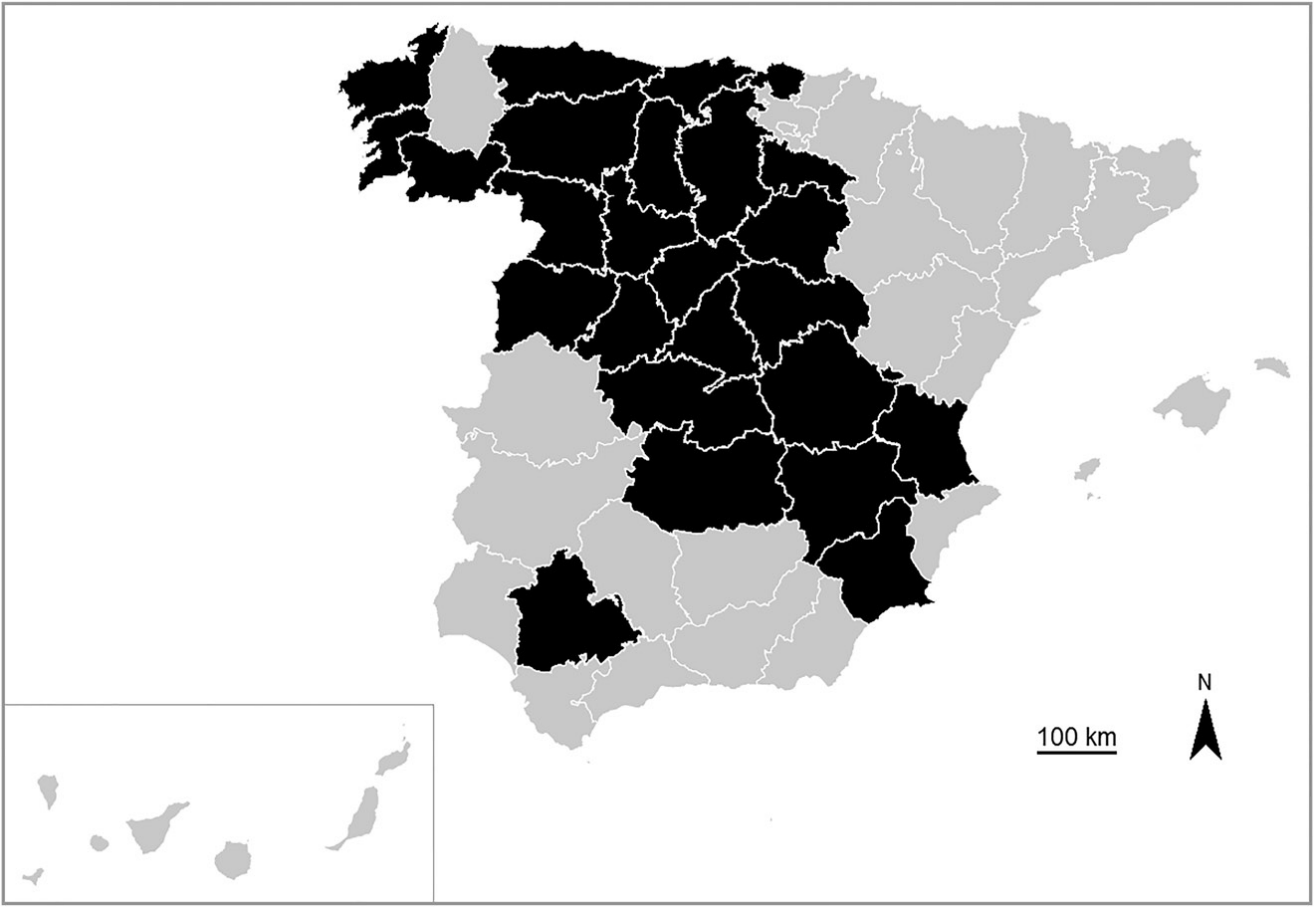
Map of Spain with the distribution of provinces where cases were recorded (in black).

This is a retrospective study based on forensic autopsies performed at the Institutes of Legal Medicine and Forensic Sciences of Biscay, Seville, Valencia and in the jurisdiction covered by the histopathology service of the National Institute of Toxicology and Forensic Sciences (NITFS) in Madrid (Spain) from 2010 to 2017 (8 years). The NITFS of Madrid is the reference centre for supplemental studies (histopathology, toxicology, microbiology, DNA, etc.) in about a third of the Spanish territory. These four forensic centres cover a total of 25 provinces in Spain with a population of 23 011 848 inhabitants (50% of the total Spanish population, 46 million people) (Figure 1).

### Study population

All SCDs in persons 1**–**35 years old during the period ranging from 1 January 2010 to 31 December 2017 (8 years) were selected. The total number of SCDs were divided into death occurred during exercise (SRSD) and death during rest, sleep or normal activities (non-SRSD). Each of these two groups was subdivided according to the cause of death into MD (primary cardiomyopathies and myocarditis) and non-MDs (atherosclerotic and non-atherosclerotic coronary diseases, valvular pathology, thoracic aortic dissection, and SCDs with structurally normal heart namely sudden arrhythmic death syndrome (SADS)).

### Autopsy procedure

According to the Spanish legislation, all cases of violent or suspicious (sudden-unexpected) deaths must undergo a medicolegal investigation with the aim of establishing the cause and the manner of death. Following the Recommendations on the Harmonization of Medico-Legal Autopsy Rules produced by the Committee of Ministers of the Council of Europe [13], a complete medicolegal investigation of the cases including death scene investigation, toxicological analysis and gross and microscopic pathological studies was performed in all SCDs, of the study population. In some cases, microbiology and postmortem genetic screening were also carried out. According to recent publications focused on investigation of SCD [14–16], histopathological studies of the hearts were performed by trained forensic pathologists with expertise in cardiovascular pathology. Macroscopic examination was performed on all whole hearts and the following measurements were taken: total heart weights comparing to expected heart weights in relation to body weights according to Kitzman et al. [17] and Vanhaebost et al. [18], and thickness of the free wall of the left and right ventricle as well as of the septum, excluding trabeculae and epicardial fat. In cases of cardiomegaly, ventricular diameters were also recorded. Coronary arteries were examined with multiple transverse cuts at 3–5 mm intervals along the course of the main epicardial arteries (with pre-decalcification, if necessary) to look for the presence of obstructive vascular disease. All hearts were documented photographically with detailed pictures of major findings. A detailed histological study of the heart was performed, and myocardial samples of the ventricles (anterior, lateral and posterior walls of left ventricle, septum and anterior and posterior walls of right ventricle) and samples of the major epicardial coronary arteries in case of moderate-severe gross stenosis were included. The examination of the conduction system, using a simplified method [19,20], was reserved to cases where no pathological findings were observed at gross or histological examination.

### Definitions

- SD was defined, according to commonly accepted criteria, in witnessed cases, as a natural death that occurs within 6 h of the beginning of symptoms in an apparently healthy subject or in one whose disease was not so severe that a fatal outcome would have been expected [21]. In cases of unwitnessed death, the definition requires that the deceased was last seen alive and functioning normally 24 h before being found dead [22].
- For the diagnosis of SCD, we included those SDs resulting from disorders affecting the different cardiovascular structures which integrity is essential for a normal heart function, e.g. coronary arteries, myocardium, cardiac valves, conduction system and intrapericardial aorta [16]. The diagnostic of the different cardiovascular entities causing SCD was performed at autopsy following internationally accepted criteria published in previous studies [10,14,16,23–25].
- Hypertrophic cardiomyopathy (HCM) when there was cardiac hypertrophy associated to extensive myocyte disarray with/without interstitial fibrosis or small intramyocardial vessel disease.
- Idiopathic left ventricular hypertrophy (ILVH) when heart weight exceeded maximum expected for body weight using reference values in absence of arterial hypertension or coronary or valve disease, and there was no myocyte disarray in microscopic examination.
- Arrhythmogenic cardiomyopathy (ACM) when myocardium of right and/or left ventricle was replaced by fibrofatty tissue associated with myocyte degenerative changes and permeable coronaries.
- Dilated cardiomyopathy (DCM) in hearts with cardiomegaly, hypertrophy, left ventricular diameter above 4 cm, and thin ventricular wall (about 10 mm), with/without fibrosis in absence of atherosclerotic coronary disease.
- Acute myocarditis was considered the cause of death when multiple foci of interstitial inflammatory infiltrate with/without myocyte necrosis were found in the myocardium. For the diagnosis, according to the Dallas criteria, it was required that the majority of inflammatory foci had necrosis.
- Cases with no significant abnormalities on cardiac macroscopic and microscopic examination and with negative toxicological screening were classified as structurally normal hearts *(“mors sine materia”*) or SADS.
- SRSD was defined as death occurring during sports activity or up to 1 h after its cessation. Competitive sports include any organized sports event that had been certified by an official, recognized sports association or authority. All other exercise related SD were categorized as non-competitive or recreational.
- Positive toxicology was defined as the presence of any substance (licit and/or illicit) upon toxicological investigation, excluding drugs administrated during cardiopulmonary resuscitation.

### Clinical information

Family history, clinical history, previous cardiac symptoms, type of sport and circumstances surrounding the death were also reviewed, when known. This information was obtained from primary care physicians and forensics reports, police reports and, when possible, from interview with the family of the deceased.

### Variables analysed

Demographic, clinical and toxicology data, cardiovascular risk factors, cause of death as well as circumstances of death (sports-related and type of sports) were analysed. Cause of death was established considering all information regarding clinical history, autopsy findings and results of all ancillary examinations. Toxicology tests results for common drugs of abuse, ethanol, psychotropic drugs and other therapeutic drugs were also noted.

### Data analysis

Continuous variables are presented as mean ± standard deviation or as median and interquartile range (P25 and P75) for non-Gaussian distributed data. Categorical variables are presented by number of cases and percentages. Differences between categorical variables were compared using χ^2^ test (Fisher's Exact Test) and Student *t* test for continuous variables. A two-tailed *P*-value <0.05 was considered statistically significant. Statistical analysis of the data was performed using SPSS for Windows version 21.0 (IBM, Armonk, NY, USA).

## Results

During the 8 years covered by the study, 645 SCD cases were investigated in 1**–**35-year-old persons in the four centres, including 75 (11.6%) SRSD and 570 (88.4%) non-SRSD. A primary or inflammatory MD was diagnosed in 145/645 (22.5%) cases.

**Figure 2.**
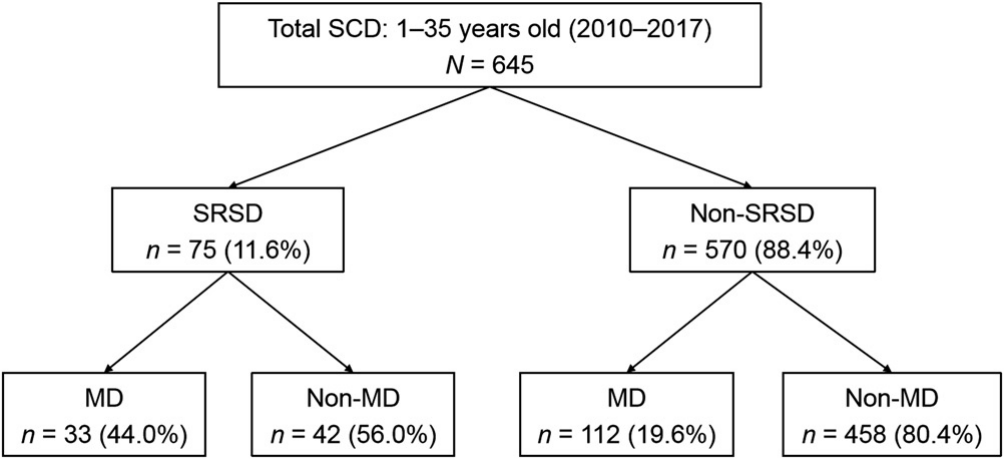
Flowchart of data collection. SCD: sudden cardiac death; SRSD: sports-related sudden death; non-SRSD: non-sports related sudden death; MD: myocardial diseases; non-MD: non myocardial diseases.

MD was diagnosed in 33/75 (44.0%) of the SRSD and in 112/570 (19.6%) of the non-SRSD cases. Conversely, 8.4% (42/500) of the SD by non-MD were sports-related as opposed to 22.8% (33/145) of the SRSD by MD. Both results indicate a strong association between SRSD and MD (*P* < 0.001) (Figure 2).

### Demographic data and causes of death

Demographic data and geographic distribution of cases are shown in Table 1.

**Table 1.** Comparison between SRSD and non-SRSD due to MD: demographic data and geographical distribution of cases.

Items	Total (*N* = 145)	SRSD (*n* = 33)	Non-SRSD (*n* = 112)
Age (years, mean ± SD)	24.2 ± 8.6	24.0 ± 7.6	24.3 ± 8.9
Sex^a^ (*n*, %)			
Male	115 (79)	33 (100)	82 (73)
Female	30 (21)	0 (0)	30 (27)
Age group (*n*, %)			
<15 yo	16 (11)	2 (6)	14 (13)
15–24 yo	49 (34)	14 (42)	35 (31)
25–35 yo	80 (55)	17 (52)	63 (56)
Service (*n*, %)			
NITFS	83 (57)	14 (43)	69 (62)
Valencia	23 (16)	9 (27)	14 (12)
Seville	27 (19)	7 (21)	20 (18)
Biscay	12 (8)	3 (9)	9 (8)

SRSD: sports-related sudden death; non-SRSD: non-sports related sudden death; MD: myocardial diseases; yo: year-old; NITFS: National Institute of Toxicology and Forensic Sciences.

a *P* < 0.05, with statistical significant.

All cases of SRSD due to MD were males while in non-SRSD the percentage was 73%. Distribution by age group was similar. Mean age was (24.0 ± 7.6) years in SRSD group (range 8–35; median 26 years) *vs.* (24.3 ± 8.9) years in non-SRSD (range 2**–**35; median 26 years). The number of cases analysed in each centre is shown in Table 1. The distribution of MD in relation to sports or non-sports activity did not show statistical significant differences between the four centres.

**Figure 3.**
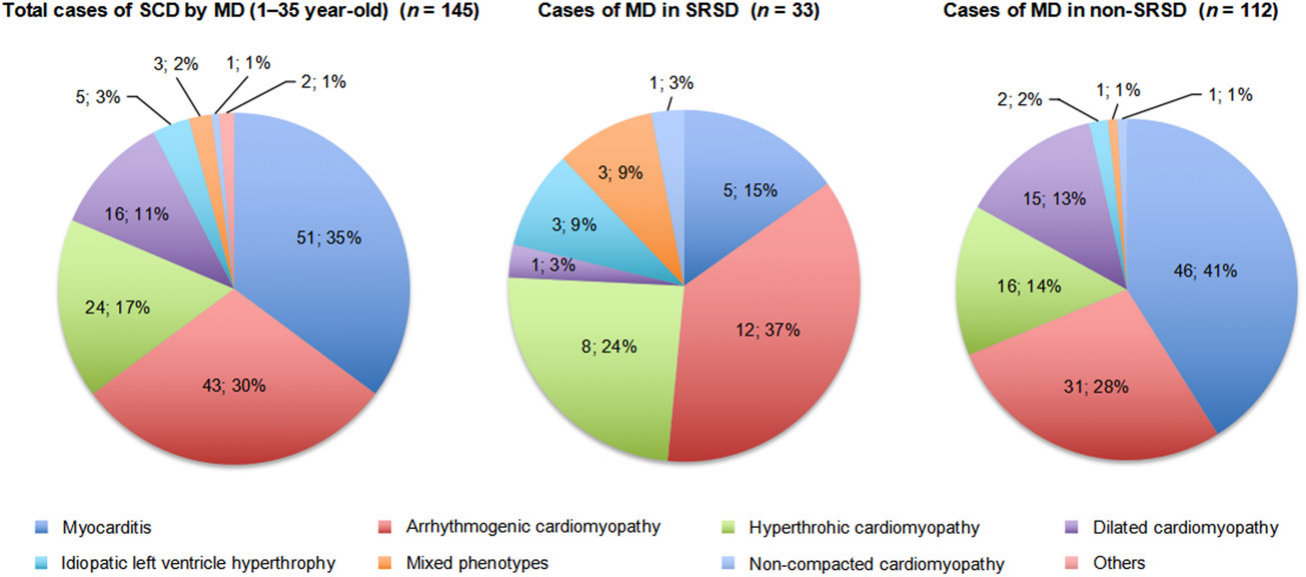
Causes of sudden cardiac death (SCD) due to myocardial diseases (MD) and their relation to exercise. SRSD: sports-related sudden death; non-SRSD: non-sports related sudden death; MD: myocardial diseases.

The main causes of SCD by MD are shown in Figure 3. Myocarditis (35%) and ACM (30%) followed by HCM (17%) and DCM (11%) were the most frequent MD. A different distribution of MD in relation to activity at the moment of death was observed (*P* = 0.01; only MD with a frequency >10% were included): SRSD was more frequent in ACM (37%) and HCM (24%), followed by myocarditis (15%) and ILVH (9%). In three cases of the SRSD group, a mixed phenotype was observed: HCM + ACM (two cases) and ACM + non-compacted cardiomyopathy (one case). On the contrary, only one DCM (3%) was associated to SD during sport activity. Our results showed that in the group of athletes, 30.7% (23/75) of the SDs were due to cardiomyopathies (ACM and HCM), which means that approximately one in three SDs in athletes under 35 years was caused by a cardiomyopathy.

### Clinical and postmortem characteristics of SRSD by MD

In nine (27%) SRSD subjects, six of them with ACM, there were some family antecedents of interest. A family history of arrhythmia was presented in four and of SD in three cases: one premature SD (defined as death of a first-degree relative <50 years of age) and two cases with SD in first-degree relatives >50 years of age. The rest of familial antecedents were ACM (brother), ischemic heart disease (father) and hypertension (father; Tables 2 and 3***)***.

**Table 2.** Clinic antecedents and type of sports in death due to MD in SRSD and non-SRSD cases.

Items	Total (*N* = 145) (*n*, %)	SRSD (*n* = 33) (*n*, %)	Non-SRSD (*n* = 112) (*n*, %)
Family antecedents	23 (16)	9 (27)	14 (12)
Personal antecedents^a^			
Cardiovascular disease diagnosed in life	11 (8)	5 (15)	6 (5)
Cardiovascular symptoms			
Yes	37 (25)	7 (21)	30 (27)
No	75 (52)	19 (57)	56 (50)
Unknown	22 (15)	2 (6)	20 (18)
Toxicological analysis^a^^,^^b^			
Positive	26 (18)	1 (3)	25 (22)
Ethanol	9	1	8
Illicit drugs	10	0	10
Therapeutic drugs	9	0	9
Negative	85 (59)	28 (85)	57 (51)
Unknown/not performed	34 (23)	4 (12)	30 (27)
Type of sport			
Recreational		28 (85)	
Competitive		5 (15)	
Sports discipline			
Football		16 (49)	
Gymnastics		5 (15)	
Running		4 (12)	
Swimming		2 (6)	
Cycling		2 (6)	
Others		4 (12)	

^a^Unknown and not performed cases were excluded from statistical analysis; ^b^*P* < 0.05, with statistical significant.

MD: myocardial diseases; SRSD: sports-related sudden death; non-SRSD: non-sports related sudden death.

**Table 3.** Cases description of 33 cases of SRSD due to MDs at autopsy.

Case No.	MD	Age (years)	Sport	Personal antecedents	Ancillary tests (genetics and microbiology)
1	ACM	28	Running	–	Not performed
2	biV-ACM	32	Football	Steroid treatment for infertility	Not performed
3	biV-ACM	32	Gymnastics	Syncope	Plakophilin (*PKP2*) mutation
4	biV-ACM	32	Gymnastics	Syncope; obesity; mother's SCD	Not performed
5	biV-ACM	18	Football	Syncope	Not performed
6	biV-ACM + non-compacted CM Mixed CM	21	Cycling	Smoker	Desmoplakin (*DSP*) mutation
7	LV-ACM	17	Football (competitive)	Arrhythmia; right bundle branch block	Negative
8	LV-ACM	32	Paddle	–	Filamin (*FLNC*) truncation
9	LV-ACM	17	Football	–	Filamin (*FLNC*) truncation
10	LV-ACM	29	Football	–	Not performed
11	RV-ACM	26	Gymnastics	Arrhythmia in treatment with β-blocker; aborted SCD at 13 years old; obesity	Plakophilin (*PKP2*) mutation
12	RV-ACM	21	Football	Arrhythmia	Plakophilin (*PKP2*) truncation
13	RV-ACM	30	Football	–	Not performed
14	HCM	16	Judo (competitive)	Glycogenosis III; cardiac hypertrophy	Not performed
15	HCM	27	Football	Non-obstructive HCM; asymptomatic	*Q327fs* mutation in the C gene protein of myosine union (in life)
16	HCM	29	Running	HCM without treatment	Missense mutation in *MYH7* gene
17	HCM	35	Running	Non-obstructive HCM	*FOHD3* and *ANK2* mutation of uncertain significance
18	HCM	13	Football	–	Not performed
19	HCM	26	Running (competitive)	–	Negative
20	HCM	24	Football	–	Not performed
21	HCM	33	Football	–	Not performed
22	ILVH	15	Football (competitive)	–	Mutation in *MYBPC3* gene; *TCAP* mutation of uncertain significance
23	ILVH	15	Cycling	–	Heterozygous polymorphisms *V158M* in *MYBPC3* (cardiac myosin-binding protein-C) and *A151A* in *TPM1* (Tropomyosin 1)
24	ILVH	22	Football	Arrhythmia	Not performed
25	HCM-ACM Mixed CM	30	Swimming	Obesity; psychomotor retardation	Negative
26	HCM + RV-ACM Mixed CM	32	Gymnastics	Apical HCM with extrasystole	Plakophilin (*PKP2*) and desmoglein (*DSG2*) mutation
27	Non-compacted CM	16	Surf	–	Not performed
28	DCM	31	Football	Cocaine dependence in treatment	Not performed
29	Myocarditis	8	Swimming	–	Microbiological screening negative
30	Myocarditis	16	Athletics (competitive)	–	Microbiology (blood and myocardium) positive borderline to herpes virus type 6
31	Myocarditis	35	Gymnastics	Hypertension	Microbiological screening not performed
32	Myocarditis	15	Football	Smoker	Microbiological screening not performed
33	Myocarditis	18	Football	–	Microbiological screening not performed

SRSD: sports-related sudden death; MD: myocardial diseases; ACM: arrhythmogenic cardiomyopathy; –: not obtained; biV-ACM: bilateral ventricular arrhythmogenic cardiomyopathy; SCD: sudden cardiac death; CM: cardiomyopathy; LV-ACM: left ventricular arrhythmogenic cardiomyopathy; RV-ACM: right ventricular arrhythmogenic cardiomyopathy; HCM: hypertrophic cardiomyopathy; ILVH: idiopathic left ventricular hypertrophy; DCM: dilated cardiomyopathy.

Personal antecedents of interest were presented in 12 (36%) cases. The MD responsible of death had only been diagnosed in life in five cases: four HCM and one glycogen storage disease type III (HCM phenocopy). Cardiovascular symptoms that could be related to the disease were present in other seven patients (six of them with ACM): aborted SCD and arrhythmia in one, syncope in three and arrhythmia in other three cases. In none of the 12 cases with ACM the disease was diagnosed in life. A cardiovascular risk factor was observed in four (12%) cases: Smoking in one and obesity (BMI >30 kg/m^2^ detected at autopsy) in three cases.

Table 2 shows the comparison between clinical data of SRSD and non-SRSD.

### Toxicological and microbiological analysis

Toxicological results were known in 29 (88%) SRSD cases, and only were positive in one (3%) with ethanol in a very low level in blood and vitreous humour (0.20 g/L and 0.24 g/L, respectively). Toxicology was positive in a major percentage (22%) of non-SRSD cases with the presence of ethanol in eight, illicit drugs (cannabis, cocaine and opiates) in 10 and therapeutic drugs in nine cases (Table 2).

Microbiological analysis was performed in 2/5 (40%) myocarditis: one was negative and in the other case herpes virus type 6 was identified (Table 3).

### Genetic analysis

Postmortem genetic studies were performed in 15/28 (54%) cardiomyopathies with positive results in 12 cases (80%): in six ACM (mutations in Plakophilin gene were the most frequent); in three HCM and in one with mixed phenotype (Table 3).

### Sports activity

The majority of SRSD were associated to a recreational sport (*n* = 28 (85%)) and only five cases were competitive athletes. The most frequent sports disciplines were football (49%), followed by gymnastics (15%) and running (12%) (Tables 2 and 3).

## Discussion

This multicentre study represents the largest series of young individuals who died suddenly due to MD during sports activity in 25 provinces of Spain representing 50% of the total Spanish population. All postmortem examinations were conducted by pathologists with experience in cardiovascular pathology using standardized diagnostic criteria. For that reason, we consider that our results are consistent and may be extrapolated to all Spanish territory.

### Type of sport

In agreement with previous studies, the majority of our cases occurred in the practice of recreational sports [3,10]. The most frequent sport disciplines affected, which coincide with the most popular in Spain, were football (49%) followed by gymnastics (15%) and running (12%). In previous Spanish series, cycling was the most frequent among older population and associated to coronary atherosclerotic disease [10]. This finding does not necessarily mean that these sports represent the highest risks for an SCD [26]. In terms of cardiovascular demand during sport at the time of the event, most SCDs occurred when moderate to high dynamic/moderate static sporting disciplines were performed [27].

### Cause of death

The frequency of MD in general and the frequency of each type of MD are very different in the published series of SD in young people. MD represented 145 of the 645 SD (22.5%). Myocarditis (51/645; 7.9%) followed by ACM (43/645; 6.7%) and HCM (24/645; 3.7%) were the most frequent MD. In the Veneto study [28], 650 SCD were studied at postmortem and the main causes were coronary atherosclerosis (18%), myocarditis (12%), ACM (10%) and HCM (9%). The percentage of myocarditis in our series was also lower than that in the study of Bohm et al. [27] in Germany where myocarditis prevailed in this age group (24%) followed by premature coronary atherosclerotic disease.

Our main finding is that the risk of SD seems to be highly related with exercise in young persons who dies due to MD. According to our results, SD by MD in young people is statistically more frequent during sports comparing to MD in non-SRSD (44.0% *vs*. 19.6%). This association was observed for ACM and HCM and not for DCM or myocarditis.

Predominance of MD in SRSD has been observed in several studies of young competitive athletes and recreational sports but different distribution of cardiomyopathies in relation to geographical origin of the series has been described [5–11]. In the USA, HCM has been the cause in 45% of SRSD [11]. In contrast, in Italy a predominance of ACM has been observed (approximately 25%) [29]. In the UK, a study on amateur athletes performed by a referral cardiovascular centre showed that the most frequent cause of SD was ILVH (30.5%) [30]. In Spain, in a series of SD during recreational sports, 31 (38%) of SRSD in persons ≤35 years old were due to MD being the main diagnosis ACM (*n* = 12), HCM (*n* = 8) and ILVH (*n* = 7) [10].

In another study in UK in a cohort of 357 SD in athletes, SRSD occurred in 61% of cases [12]. The authors observed that ACM was the strongest independent predictor of SCD during exercise, followed by ILVH and idiopathic left ventricular fibrosis (ILVF) [12]. In our series ACM was the most common entity in SRSD (37%).

However, different findings have been observed in other studies. In Switzerland, a lower percentage of MD in SRSD among persons aged 10–39 years was observed (15/52 = 28%) [31]. HCM was the most frequent (*n* = 9) [31]. In Denmark, of the 35 SRSDs (range 12–49 years old) with autopsy, MD has a low frequency (9/35 = 26%). ACM with five cases was the main MD diagnosed [32]. In Germany, there were 37 SRSDs, of which 16 (43%) were MD, but 11 of them were due to myocarditis and hereditary cardiomyopathies were diagnosed only in five (13%) cases [27]. In Norway, 23 SRSDs in young adults aged 15–34 years were studied. The frequency of MD was 26%. There were five myocarditis and only one case of HCM [9]. In Ireland, of the 11 SRSD in persons <35 years old, only one case was due to HCM [33]. In Southern China, cardiomyopathies were the most common cause of death (31.2%) in those <35 years old (ACM = 4; restrictive cardiomyopathy = 3; DCM = 2; and HCM = 1). Viral myocarditis was diagnosed in only one case [34].

Other studies showed a lower proportion than ours (23%) of young subjects who died by MD in relation to sport activity. Chappex et al. [35] reported in Lausanne (Switzerland) 59 SDs by MD in persons 10–50 years old and only nine (15%) died during sport; the percentage was high in ACM (33%) but not in HCM (12%) and myocarditis (12%). In a similar study in Switzerland, of 131 SDs by MD in persons aged 10–39 years old, only 15 (11%) were sports-related [31].

The reasons for the discrepancy are unclear but it could be explained by different factors: i) regional differences in population characteristics; ii) different cohort (age range and type of population); iii) different methodology in the studies (not always postmortem examination is performed); iv) different diagnostic criteria; and v) experience on SCD of the forensic pathologists who perform post-mortem examination of the heart.

Interpretation of some causes of death can be difficult in forensic pathology. In order to improve the diagnosis of SCD, the Association for European Cardiovascular Pathology (AECVP) has updated the guidelines for autopsy investigation of SCD considering a degree of certainty in the cardiovascular substrates found at postmortem examination. On the other hand, the establishment of regional multidisciplinary expert networks could be an interesting approach for standardization of diagnostic criteria [16].

### Gender

Male gender seems to be an independent risk factor for SRSD due to MD. In our study it was striking that all cases were male (in non-SRSD this percentage was 73%). Predominance of males has been described in all previous studies, with percentages ranging from 70%–90% [3,10,27,30,31,34].

This difference could be explained due to several factors: i) higher incidence of SCD, and especially MD, in males; ii) more males are engaged in competitive sports; and iii) a harder mode of physical activity and training in males, especially in the field of non-elite competitive sport [10,27]. The Spanish population survey shows that 53.5% of the population over 15 years old practice sports (86% with great intensity); 50% of males and 42% of females practice sports in a weekly basis and the mean of sports time per week is higher in males than in females (440.7 min *vs*. 269.2 min) [36].

### Clinical history and screening

Preparticipation screening offers the potential to identify asymptomatic athletes with potentially lethal cardiovascular abnormalities and to prevent SD through disqualification from competitive sports [29,37–41].

One of the main findings of the present investigation was the relatively high percentage of patients with symptoms before occurring of the SD event. In 36% of our cases, a clinical symptom suggestive of cardiovascular disease, mainly arrhythmia and syncope, was present in life, which is in agreement with the series of Chappex et al. [35] (36.4% symptomatic) but contrast with other series like Finocchiaro et al. [12] (19% symptomatic). This finding outlines the importance of the implementation of the preparticipation screening in Spain, including history, clinical examination and resting 12-lead ECG, that is mandatory in other European countries such as Italy and Switzerland [29,31]. Nevertheless, we understand the difficulty of this screening in general population performing recreational sport. In our series four patients diagnosed of HCM and one diagnosed of glucogenosis (HCM phenocopy) died during sports that alert about criteria for restriction in sport activity in these patients. On the other hand, no one of the 12 youngs’ death due to ACM was diagnosed in life, although one had suffered an aborted SCD and three had antecedents of syncope. More efforts have to be made in order to take a better clinical diagnosis of this entity still unknown in many aspects, especially the left variant. In these cases, the disqualification from sports should be recommended. On the other hand, the immediate access to an automatic external defibrillator (AED) in public spaces and sport facilities could be potentially life-saving for cases that are not detected during preparticipation screening [12].

Four (12%) subjects had at least one cardiovascular risk factor, mainly obesity, which means that the likelihood of a cardiovascular event was certainly increased.

Genetic screening represents an important tool to support the forensic investigation and implies great progress for relatives of young SCD victims facilitating adequate risk stratification and genetic counselling [42]. Especially in SRSD due to MD genetic screening is mandatory because sport is a widely known modifier that can influence the phenotypic expression of these pathologies. The role of genetic test is especially relevant in cases of mixed phenotype, observed in three subjects of this series: in one a desmoplakin mutation was detected, in other plakophilin and desmoglein mutation was detected and in the last one was negative.

## Study limitations

Due to the retrospective nature, clinical information as well as family and personal clinical history is missing in some cases. The NITFS in Madrid is a referral centre that could not receive all cases of SRSD from its territory, introducing a potential referral bias with underestimation of cases. However, we consider that our results represent a large series of SRSD and may be a good representation of the SRSD in young people in Spain.

## Conclusion

In Spain, SRSD in young people due to MDs occurs in males who perform a recreational activity. Compared with control group we observed a strong association between MDs and exertion. One in three SRSDs are due to cardiomyopathy, especially ACM, which reinforces the need for preparticipation screening to detect these pathologies also in recreational sport athletes. Further studies are needed to understand the causes and circumstances of SD to facilitate the development of preventive strategies.
